# An educational leaflet improves response to invitation for screening for arthritis in patients with psoriasis in primary care, but only in practices in the most deprived areas

**DOI:** 10.1007/s10067-016-3503-7

**Published:** 2016-12-15

**Authors:** Laura C. Coates, Laura Savage, Robin Waxman, Dennis G. McGonagle, Anna R. Moverley, Philip S. Helliwell

**Affiliations:** 1 Leeds Institute of Rheumatic and Musculoskeletal Medicine, University of Leeds, 2nd Floor, Chapel Allerton Hospital, Harehills Lane, Leeds, LS7 4SA UK; 2 Leeds Musculoskeletal Biomedical Research Unit, Leeds Teaching Hospitals NHS Trust, Leeds, UK

**Keywords:** Patient education, Psoriatic arthritis, Screening

## Abstract

This study hypothesises that an educational leaflet about psoriatic arthritis (PsA) will improve psoriasis patients’ attendance for screening for PsA. A random sample of patients ≥18 years old with a coded diagnosis of psoriasis and no diagnosis of PsA, rheumatoid arthritis or ankylosing spondylitis were identified from five GP surgeries in Yorkshire, UK. Patients were randomised 1:1 to receive study information alone or with the educational leaflet, with an invitation to attend for a screening examination by a dermatologist and rheumatologist. Nine hundred thirty-two invitation packs were sent to recruit 191 (20.5%) participants. One hundred sixty-nine (88.5%) had current or previous psoriasis and 17 (10.1%) had previously undiagnosed PsA. The estimated prevalence of PsA was 18.1% (95% CI: 16.2, 20.1%).

The response rate was lower than expected and was not significantly higher when patients received the educational leaflet (22.8 vs 18.3%, *p* = 0.08). Response rates varied by practice (14.7 to 30.6%). However, deprivation scores for each practice revealed a significant increase in response with the leaflet for deprivation decile of 3 (*p* < 0.001) but no significant differences in the other practices. An educational leaflet about PsA improves attendance for screening in primary care, but only in those practices with higher levels of socioeconomic deprivation.

## Introduction

Psoriatic arthritis (PsA) manifests clinically in several ways including arthritis, enthesitis, dactylitis, axial disease, skin and nail involvement. The majority of people with this condition have pre-existing psoriasis [1], but studies have shown that there are many cases of established PsA which remain unidentified for some time, despite an established diagnosis of psoriasis [2]. Possible causes of this are patients’ lack of understanding of the link between the skin and arthritis, and the lack of musculoskeletal expertise in primary care and, for those referred with their skin problem, treating dermatologists. It follows that a simple method of screening for psoriatic arthritis in people with psoriasis would prevent unnecessary suffering and enable earlier treatment of this potentially disabling disease [3]. Indeed, recent consensus guidelines from the UK for managing psoriasis (both the Scottish Intercollegiate Guidelines Network (SIGN) guidelines for psoriasis and psoriatic arthritis [4] and the National Institute for Health and Care Excellence (NICE) guidelines for management of psoriasis [5]) recommend annual screening for PsA amongst patients with psoriasis both in primary and secondary care.

However, the response to screening is not optimal. In the comparison of three screening tools to detect psoriatic arthritis in patients with psoriasis (CONTEST) study, comparing screening questionnaires in secondary care dermatology clinics, there was only a 70% response rate to the questionnaires and of those subsequently contacted for a rheumatological assessment, only 61% of these attended. This was despite a patient information sheet explaining why the study was being conducted.

As noted above, one of the limitations of screening is that many patients are unaware of the risk of PsA and the importance of screening for it. In primary care, many patients with mild psoriasis are not under regular follow-up for psoriasis with either their GP or a dermatologist, and therefore there is no opportunity to educate them about PsA.

In other fields in rheumatology, patient education leaflets have been developed to inform patients of particular health risks with some evidence of improvement in patient awareness and attendance for investigation and treatment [6]. The primary aim of this study is to test whether educational material explaining the risk of PsA will improve attendance of patients with psoriasis for screening.

## Materials and methods

**Fig. 1:**
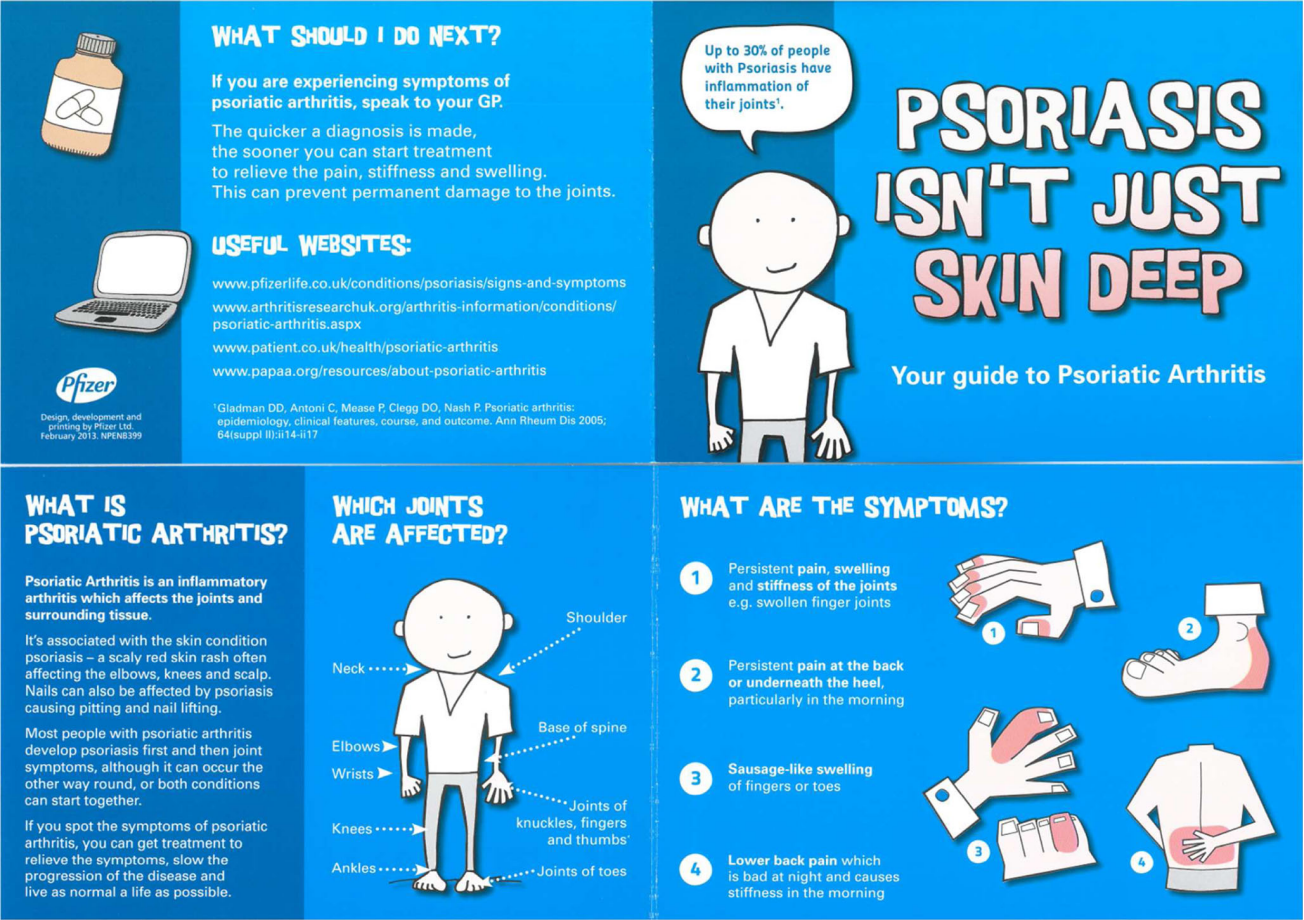
Educational leaflet sent with study information

Ethical approval was granted by the National Research Ethics Committee, UK. Patients with psoriasis were identified from five primary care practices across Yorkshire with varied socioeconomic backgrounds. Each practice performed a database search to identify potential subjects. Patients were eligible for participation if they were aged 18 years or over, had a diagnostic label of psoriasis (by read code M161, x506Y, M16Y) but did not have a coexistent diagnosis of psoriatic arthritis (read code M160), ankylosing spondylitis (read code N100) or rheumatoid arthritis (read code N40). A random sample from each practice was taken and information about the study was posted to those patients from November 2013 to November 2014. Patients were randomised 1:1 to receive the study information alone or the study information with an educational leaflet about psoriatic arthritis (Fig. 1).

The educational leaflet was a small folded card with stylised graphics depicting the common ways in which PsA can present, including peripheral arthritis, dactylitis, enthesitis and spinal disease. All patients were asked to return a reply slip if they were willing to attend one study visit at their general practitioner’s (GP) surgery for assessment by a visiting dermatologist and a visiting rheumatologist. Clinics were held in the evening to aid attendance. The dermatologist and rheumatologist were blind to the intervention.

At the study visit, following informed, written consent, patients were asked to complete a questionnaire booklet including the Psoriasis Epidemiology Screening Tool (PEST) [7] and CONTEST screening questionnaires [8], PsA quality of life (PsAQoL) [9], dermatology life quality index (DLQI) and health assessment questionnaire (HAQ). They were then reviewed independently by a dermatologist (LS) and a rheumatologist (LCC, ARM or PSH). Dermatology assessment confirmed the presence of psoriasis and assessed severity of skin and nail involvement. Rheumatology assessment included examination of any arthritis, entheseal tenderness and dactylitis to make a diagnosis of PsA where appropriate.

## Statistics

To compare the response rates according to the provision of the educational leaflet, it was assumed that a 20% difference in response rate (50% response without leaflet, 70% response rate with leaflet) would be of clinical significance. Given this 20% difference, an alpha level of 0.05, and a beta of 0.9, using a two-sided test, the estimated sample size is 124 per group, 248 in total. This represents the total number of screening invites that need to be sent out to patients identified by the practices.

A secondary aim of this study was to validate the CONTEST screening questionnaire which required a minimum sample size of 191 patients attending for examination. Therefore, the study aimed to recruit a total of 191 patients to be examined with a minimum of 248 packs distributed.

Response rates to the leaflet were compared using a chi-squared test. Analysis was done using IBM’s SPSS Statics Software.

## Results

A total of 932 packs were sent out from the five GP practices to recruit 191 participants who agreed to attend for assessment. The response rate to the study invitation was lower than predicted at 20.5%. Of the patients attending for assessment, 169 (88.5%) were found to have current or previous psoriasis. Using physician diagnosis, 17 (10.1%) were found to have previously undiagnosed PsA, 90 (53.3%) were found to have another musculoskeletal complaint and 62 (36.7%) had no musculoskeletal problems. Using data from the practices and correcting for misdiagnosis of psoriasis, the estimated prevalence of PsA was 18.1% (95%CI 16.2–20.1%).

Overall, the response rate was lower than predicted and was not significantly higher when patients received the educational leaflet (22.8 vs 18.3%, *p* = 0.088). There was a marked variation in response rates between the five GP practices involved (14.7 to 30.6%). When considering each practice individually, response rates, stratified by leaflet provision, are shown in Table 1.

**Table 1:** Response rates by leaflet provision for individual GP practices

Practice	Deprivation index	Information given	No. of packs sent	Response rate (%)	Pearson chi squared	*p* value
A	10	Leaflet	54	29.6	0.044	0.835
		No leaflet	54	31.5		
B	3	Leaflet	46	30.4	13.21	<0.001
		No leaflet	54	3.7		
C	10	Leaflet	150	21.3	0.001	0.976
		No leaflet	151	21.2		
D	10	Leaflet	136	21.3	0.109	0.741
		No leaflet	137	19.7		
E	7	Leaflet	75	18.7	1.92	0.166
		No leaflet	75	10.7		

Baseline characteristics of the leaflet and no leaflet groups are shown in Table 2.

**Table 2:** Baseline characteristics by leaflet

	No leaflet (*n* = 86)	Leaflet (*n* = 105)	*p*
Age	59 (55.62)	58 (55.61)	0.82
Sex (male)	56%	45%	0.15
Ps duration (years)	27 (23.31)	27 (24.31)	0.96
Diagnosis			0.35
PsA	8 (9%)	9 (9%)	
Not MSK	36 (42%)	34 (32%)	
OA/mechanical	32 (38%)	51 (48%)	
Other MSK	10 (11%)	11 (11%)	
PSO			0.37
Current	69 (80%)	75 (71%)	
Previous	9 (11%)	16 (15%)	
Not PSO	8 (9%)	14 (13%)	
PSO symptoms on the day			0.77
No	36 (42%)	47 (45%)	
Yes	50 (58%)	58 (55%)	
PASI score	4.0 (2.9, 5.1)	2.3 (1.6, 3.0)	0.01
mNAPSI score	6.6 (4.2, 9.0)	5.1 (3.2, 7.0)	0.34
Enthesis score	1.1 (0.5, 1.7)	1.8 (0.9, 2.6)	0.24
Dactylitis present	1 (1%)	0	0.27
Tender joint count	1.7 (1.0, 2.4)	3.5 (1.7, 5.3)	0.09
Swollen joint count	0.4 (0.1, 0.6)	0.8 (0.3, 1.3)	0.14
HAQ	0.2 (0.1, 0.3)	0.3 (0.2, 0.6)	0.11
PsAQoL	3.3 (2.2, 4.4)	4.2 (3.0, 5.4)	0.63
DLQI	3.6 (2.7, 4.5)	3.5 (2.6, 4.4)	0.87
Contest	3.7 (3.0, 4.4)	3.7 (3.0, 4.5)	0.44
Contest joints reported (mean)	4.8 (3.8, 5.9)	5.8 (4.7, 6.8)	0.36

*PsA* psoriatic arthritis, *MSK* musculoskeletal, *PSO* psoriasis, *PASI* psoriasis area and severity index, *mNAPSI* the modified nail psoriasis severity index, *HAQ* health assessment questionnaire, *PsAQoL* the psoriatic arthritis quality of life measure, *DLQI* dermatology quality of life index

Socioeconomic data of the registered patients at each practice were obtained from National Statistics on deprivation with each practice given a decile score (range 1–10 where level 1 represented the most deprived area and level 10 the least deprived—Public Health England, National General Practice Profiles). In our study, one practice had a deprivation score of 3, one had a score of 7 and the rest had a score of 10. Analysing the impact of the leaflet on response rates by deprivation showed that there was a significant increase in response with the leaflet for the practice with a deprivation decile of 3 (response rate 30.4 vs 3.7%, *p* < 0.001), but there was no significant difference identified in the other practices. In the practice with the deprivation index of 7, there was a numerical difference between the response rates (18.7 vs 10.7%) but it did not reach significance. Only 150 packs were sent out at this practice.

## Discussion

This study did not identify a significant improvement in attendance for PsA screening in the entire cohort but within the practice with the lowest deprivation index; there was a marked difference in the proportion attending for screening. In the practice with the deprivation index of 7, there was a suggestion of a difference but it did not reach significance (18.7 vs 10.7%, *p* = 0.166). Given that there were only 150 packs sent out at this practice, this may represent a type II error.

The response rate was lower than predicted at around 20%. This is significantly lower than response rates in previous secondary care settings, but this was an unsolicited postal invitation, albeit from their primary care provider, rather than an invitation from their treating dermatologist, if the patient had a secondary care provider. This may have introduced a type II error as the sample size originally assumed a 50% response rate. However, in the overall sample, the difference in response was not large enough to suggest utility of the leaflet for general use. The least deprived practices (A, C and D) did not show any difference in response with the leaflet and also showed the higher response rates of 20–30%. There was a trend towards a difference in practice E with a deprivation index of 7 and a strong effect with the leaflet in practice B which had the lowest deprivation index of 3. This suggests that the leaflet may play a useful educational role in low socioeconomic areas. In such areas, attendance for health screening is traditionally poor, and giving this information in an easy to read, cartoon format at ‘opportune’ moments, such as consultations, may improve identification of psoriatic arthritis in patients with psoriasis. The response rate without the leaflet was only 3% but increased with the leaflet to 30%, similar to maximum response rate at any of the practices.

The assumption that screening to identify undiagnosed cases of PsA is worthwhile has not been formally assessed but is based on observational studies in which shorter disease latency at diagnosis is associated with better outcomes [10–12]. Although the National Institute for Health and Clinical Effectiveness (NICE) has recommended annual screening for PsA [5], anecdotal evidence suggests this has not been widely adopted (personal communication from the Psoriasis Association). Clearly both patients and health providers in both primary and secondary care need more education about the risks associated with undiagnosed (and by implication untreated) PsA. The matter is further complicated by the fact that many patients with relatively mild psoriasis do not regularly visit a health professional in either primary or secondary care, perhaps being happy to self-medicate this problem. The lack of association between the severity of the skin disease and the severity of the musculoskeletal disease does not mean that mild psoriasis means mild PsA.

The main strength of this study is that it was conducted in a real life, community-based, screening program using a validated screening questionnaire. The study was also able to access a diverse range of communities varying widely in socioeconomic status. Further, all patients had a full examination by a rheumatologist and a dermatologist, in the primary care setting. A weakness was the poor overall response to invitation for screening. As this was far below the expected response rate, it is likely that this study was underpowered, as the sample size was based on a much higher response rate (50/70% predicted for ‘no leaflet’/‘leaflet’ against 18/23% actual study figures). The study is also not able to explain why there was a higher response to screening with the leaflet in the poorer socioeconomic areas but may reflect the educational value of the leaflet in this less well-educated population.

In conclusion, screening for musculoskeletal disease in people with psoriasis is important in order to reduce long-term morbidity. It is likely that education of patients and physicians is likely to have the most impact: the addition of a simple leaflet may help, particularly if given in person by a health care practitioner. The leaflet as an educational tool is likely to be of more benefit in lower socioeconomic areas.
